# Successful repeated uterine artery embolization in postpartum hemorrhage with disseminated intravascular coagulation: a case report and literature review

**DOI:** 10.1186/s12884-021-04191-9

**Published:** 2021-10-22

**Authors:** Shunya Sugai, Taro Nonaka, Kana Tamegai, Tatsuhiko Sato, Kazufumi Haino, Takayuki Enomoto, Koji Nishijima

**Affiliations:** 1 Departments of Obstetrics and Gynecology, Niigata University Medical and Dental Hospital, 1-757 Asahimachi-dori, Chuo-ku, 951-8510 Niigata, Japan; 2 Radiology and Radiation Oncology, Niigata University Medical and Dental Hospital, Niigata, Japan

**Keywords:** Disseminated intravascular coagulation, Gelatin sponge, N-butyl cyanoacrylate, Postpartum hemorrhage, Uterine artery embolization

## Abstract

**Background:**

Postpartum hemorrhage (PPH) is a potentially fatal condition requiring urgent and appropriate intervention. Uterine artery embolization (UAE) has a high hemostatic capacity for PPH, but it may fail. Disseminated intravascular coagulation (DIC) has been reported as a risk factor associated with the failure of UAE.

**Case presentation:**

A 37-year-old primigravida with dichorionic diamniotic twins and placenta previa underwent cesarean section. The blood loss during surgery was 4950 mL. Hemostasis was achieved using an intrauterine balloon tamponade device. However, she lost a further 2400 mL of blood 5 h after surgery. We embolized both uterine arteries using gelatin sponges and confirmed hemostasis. She was suffering from DIC and received ample blood transfusions. However, a further 1300 mL of blood was lost 18 h after surgery and we performed repeated UAE, with complete recanalization of the uterine arteries on both sides and re-embolization with gelatin sponges. Her DIC was treated successfully by blood transfusions at this time, and she showed no further bleeding after the repeated UAE.

**Conclusions:**

DIC is a risk factor for the failure of UAE. Repeated UAE may be effective after sufficient improvement of the hematological status in patients with PPH and DIC.

## Background

Postpartum hemorrhage (PPH) is a major cause of maternal mortality and morbidity [1]. Causes of PPH include uterine atony, placenta previa, cervical tears, coagulation disorders, and retained parts of the placenta. Successful management of PPH can be achieved by identification of the cause and rapid treatment. PPH is initially managed with less-invasive treatments, including uterotonic agents and tranexamic acid. If uterine bleeding persists despite these interventions, mechanical and surgical interventions should be initiated. An internal balloon tamponade device can provide hemostasis by directly compressing the placental bed. Uterine compression sutures stop uterine bleeding by external pressure on the uterus. Additionally, ligation of the uterine artery and internal iliac artery interrupts the flow of blood vessels to the uterus. Peripartum hysterectomy is performed when all of these other treatment options are exhausted [2].

Uterine artery embolization (UAE) is frequently used as an alternative treatment to other interventions. The effectiveness of UAE is high and a recent review reported a 90 % success rate [3]. However, UAE is not always successful, and various factors, including disseminated intravascular coagulation (DIC), have been associated with the failure of UAE [4]. We report the success of repeated UAE in a patient with PPH and DIC.

## Case presentation

The patient was a 37-year-old primigravida who had no medical history. Her pregnancy was achieved using fertility treatment and she was carrying dichorionic diamniotic twins. She also had placenta previa. Both of the patient’s fetuses were well developed, and their condition was good.

She underwent a cesarean section at 35 weeks of gestation because she complained of frequent uterine contractions and we were concerned about heavy genital bleeding. She delivered healthy male and female neonates (2426 g, Apgar score of 8 at 1 min; and 2196 g, Apgar score of 8 at 1 min) through a transverse incision of the uterine body, to avoid making an incision in the low placenta. She experienced extensive intraoperative bleeding owing to uterine atony and placenta previa, with 4950 mL of blood loss. We inserted an intrauterine balloon tamponade device and sutured the uterine incision in two layers, with successful hemostasis. We transfused 6 units of red blood cells (RBCs) and 4 units of fresh frozen plasma (FFP) during surgery.

**Fig. 1:**
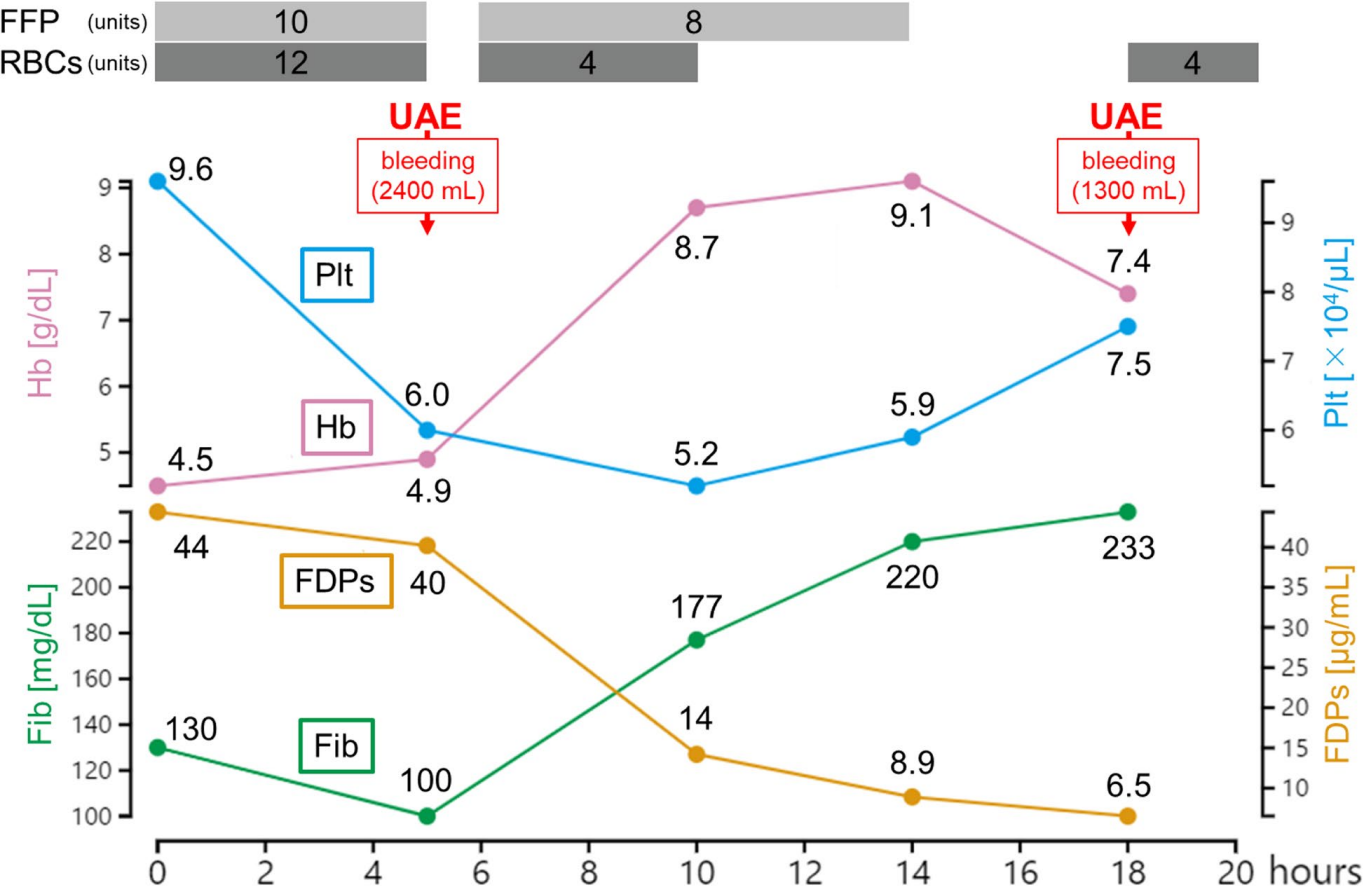
The patient’s postoperative course. FDPs: fibrin degradation products, FFP: fresh frozen plasma, Fib: fibrinogen, Hb: hemoglobin, Plt: platelets, RBCs: red blood cells, UAE: uterine artery embolization

The patient’s postoperative course is shown in Fig. 1. Postoperative blood tests showed a hemoglobin concentration of 4.5 g/dL, platelet count of 9.6 × 10^4^/µL, fibrinogen concentration of 130 mg/dL, and fibrin degradation product (FDP) concentration of 44 µg/mL. Her DIC score was 4 points according to the diagnostic criteria of the International Society for Thrombosis and Hemostasis (ISTH). Her systolic blood pressure was 123 mmHg and her pulse rate was 90 beats/min. Her hemodynamics were stable, her uterus was contracting well, and there was no bleeding. We performed blood transfusions with 12 units of RBCs and 10 units of FFP.

However, she experienced further excessive bleeding 5 h after surgery and received 10 U oxytocin, 0.2 mg methyl ergometrine, and 1 g tranexamic acid intravenously. However, these treatments were ineffective, and her blood loss was 2400 mL. Blood tests showed a hemoglobin concentration of 4.9 g/dL, platelet count of 6.0 × 10^4^/µL, fibrinogen concentration of 100 mg/dL, and FDP concentration of 40 µg/mL. According to the ISTH diagnostic criteria, her DIC score was 5 points, which indicated overt DIC. We performed UAE and embolized both uterine arteries with gelatin sponges. Postembolization arteriography showed occlusion of both uterine arteries, and her bleeding subsided. We continued blood transfusions, and blood tests at 10 and 14 h after surgery showed an improvement in DIC.

**Fig. 2:**
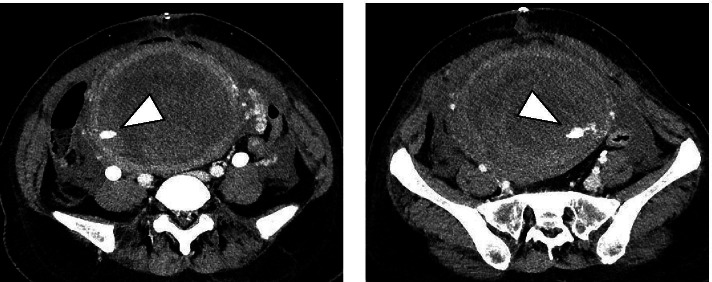
Contrast-enhanced computed tomography images. A computed tomography scan performed during the arterial phase shows extravasation from the bilateral uterus body (arrowheads)

However, the patient experienced further excessive bleeding 18 h after surgery. Dynamic computed tomography was performed to identify the source of bleeding, and extravasation from both uterine arteries to the lumen of the uterus was observed in the arterial phase **(**Fig. 2**)**. There were no findings indicating retroperitoneal hematoma or uterine rupture. Her blood loss was 1300 mL. Blood tests showed a hemoglobin concentration of 7.4 g/dL, platelet count of 7.5 × 10^4^/µL, fibrinogen concentration of 233 mg/dL, and FDP concentration of 6.5 µg/mL, and her DIC score was 1 point. Her DIC was treated successfully by blood transfusions. Her hemodynamics were stable.

**Fig. 3:**
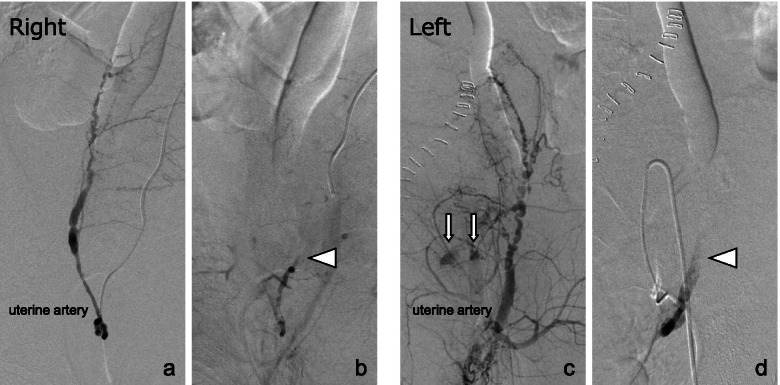
Arteriography before and after uterine artery embolization. **a** The right uterine artery was completely recanalized. **b** The right uterine artery was completely embolized using a gelatin sponge (arrowhead). **c** The left uterine artery was completely recanalized and showed extravasation (arrow). **d** The left uterine artery was completely embolized using a gelatin sponge (arrowhead)

We decided to perform repeated UAE. The uterine arteries on both sides were completely recanalized. We found extravasation only in the left uterine artery. We re-embolized both uterine arteries with gelatin sponges, and postembolization arteriography showed occlusion of both uterine arteries **(**Fig. 3**)**. No further bleeding was observed.

The total estimated blood loss throughout the process was approximately 9000 mL. The patient received 26 units of RBCs and 22 units of FFP. The patient’s condition after UAE was good and she was discharged on the 7th day postoperatively.

### Discussion and conclusion

UAE is the preferred treatment for refractory PPH because of its high efficacy and safety, low invasiveness, and preservation of fertility. The success rate of temporary hemostasis is reported to be 99 %, and the success rate with no additional treatment is 90 % [3]. However, while UAE is usually highly effective, it can fail. Little is known about the risk factors associated with the failure of UAE.

We reviewed the factors associated with the failure of UAE. We identified articles in PubMed using the following search string: (Uterine Artery Embolization[MeSH] OR pelvic-embo* OR artery-embo* OR arterial-embo*) AND (postpartum hemorrhage[MeSH]). All included articles were peer reviewed, were published in English from 1 to 2000 to 31 August 2021, and also included relevant articles identified by manual searching. We extracted information on factors associated with the failure of UAE from the manuscripts. We excluded studies where the definition of success or failure was ambiguous and no significant difference was found in any factors. The 15 included articles are shown in Table 1 [4–18].

**Table 1:** Summary of risk factors associated with uterine artery embolization failure in patients with postpartum hemorrhage

No	First author	Year published	No. of cases	Success	Failure	Risk factors by univariate analysis	Notes
1	Shim [5]	2006	49	42 (86 %)	7 (14 %)	(1) abnormal placentation	No multivariate analysis.
2	Touboul [6]	2008	102	59 (58 %)	43 (42 %)	(1) cesarean section, (2) hemodynamic shock	These two factors were significant in multivariate analysis.
3	Kirby [7]	2009	43	34 (79 %)	9 (21 %)	(1) the existent of extravasation	No multivariate analysis.
4	Sentilhes [8]	2009	100	89 (89 %)	11 (11 %)	(1) blood loss > 1500ml, (2) transfusion > 5 RBC units	No multivariate analysis.
5	Ganguli [9]	2011	66	63 (95 %)	3 (5 %)	(1) transfusion requirement after UAE	No multivariate analysis.
6	Bros [10]	2012	148	136 (92 %)	12 (8 %)	(1) primiparity, (2) coagulation disorders (3) anatomic variant of the uterine arterial vasculature	These three factors were significant in multivariate analysis.
7	Lee [11]	2012	251	217 (86 %)	34 (14 %)	(1) cesarean section, (2) DIC, (3) transfusion > 10 RBC units	(2) and (3) were significant in multivariate analysis.
8	Poujade [12]	2012	98	90 (92 %)	8 (8 %)	(1) placenta accreta spectrum, (2) biologic factor (hemoglobin level, prothrombin time, fibrinogen level), (3) transfusional factor (RBC and FFP transfusion, the number of packed RBC units)	No multivariate analysis.
9	Kim [4]	2013	257	233 (91 %)	24 (9 %)	(1) hemodynamic instability, (2) hemoglobin level < 8 g/dL (3) DIC, (4) the existent of extravasation	Only DIC was significant in multivariate analysis.
10	Cheong [13]	2014	117	103 (88 %)	14 (12 %)	(1) DIC, (2) transfusion > 10 RBC units (3) embolization both uterine and ovarian arteries	(2) and (3) were significant in multivariate analysis.
11	Zhang [14]	2015	68	53 (78 %)	15 (22 %)	(1) hemodynamic instability, (2) hemoglobin level < 9.5 g/dL (3) DIC	No multivariate analysis.
12	Tanahashi [15]	2017	57	43 (75 %)	14 (25 %)	(1) uterine height, (2) systolic blood pressure (3) hemoglobin level	(1) and (2) were significant in multivariate analysis.
13	Lai [16]	2017	33	24 (73 %)	9 (27 %)	(1) maternal age, (2) blood loss, (3) platelet count (4) history of miscarriage	(1) and (3) were significant in multivariate analysis.
14	Aoki [17]	2018	33	28 (85 %)	5 (15 %)	(1) retained placental tissue, (2) type of ovarian artery (3) the existent of extravasation	No multivariate analysis.
15	Ueshima [18]	2018	63	45 (71 %)	18 (29 %)	(1) uterine arteriography classification, (2) placenta disorders	Only uterine arteriography classification was significant in multivariate analysis.

Kim et al.’s study [4] included the largest number of cases and identified DIC as an independent failure factor for UAE. Other studies also reported that DIC and coagulation disorders were risk factors for the failure of UAE [10–14]. However, the definitions of DIC differed among these reports. Kim et al., Cheong et al., and Zhang et al. [4, 13, 14] used the ISTH definition of DIC, while Lee et al. [11] described the diagnostic criteria in their report, and Bros et al. [10] did not define coagulation disorders. To use DIC as a clinically useful factor, it should be defined according to the ISTH criteria, rather than using a vague term such as coagulation disorders.

In the current case, we used the ISTH definition of 5 points for the first UAE and 1 point for repeated UAE, which indicated an improvement in DIC. We consider that this improvement in DIC led to the success of repeated UAE.

Some studies [4, 8, 9, 11–16] have suggested that estimated blood loss, hemoglobin concentrations, and total blood transfusion volume might be associated with the failure of UAE. The amount of blood loss during the perinatal period may be underestimated [19]. Additionally, the volumes of blood loss and blood transfused are based on the final state of the patient, and are not useful for predicting the failure of UAE. We believe that DIC results in an increase in blood loss and blood transfused. DIC is easily diagnosed using tests and may be a useful clinical predictor of UAE failure. Hemodynamic instability has also been reported as a factor for the failure of UAE [4, 6, 14]. Therefore, hemodynamic stabilization and treatment of DIC using adequate fluids, RBCs, FFP, and platelet transfusions are important to increase the success rate of UAE.

A limitation of this review is that each study had a different success rate. There are some important differences between the studies, including the cause and severity of PPH, and the presence or absence of DIC. A multicenter prospective study with a larger sample size with unified conditions is required to further clarify the factors associated with the failure of UAE.

We considered the possible reasons for the low success rate of UAE for PPH with DIC. Gelatin sponges are generally the preferred embolic material. A gelatin sponge is absorbed within approximately 2–6 weeks and is thus classified as a temporary embolic material. A gelatin sponge achieves vascular occlusion by filling the blood vessel, resulting in physical stagnation of blood flow and the formation of a thrombus around the sponge [20]. Therefore, the ability of the gelatin sponge to cause an embolism depends on the patient’s coagulation ability, suggesting that the material is more likely to fail when used in patients with DIC.

DIC is a common complication of PPH, and untreated DIC can lead to further massive uncontrolled bleeding. Successful UAE in patients with DIC requires their DIC to be corrected first, or an embolic material that does not depend on the patient’s coagulation ability, such as N-butyl cyanoacrylate (NBCA), needs to be used. NBCA achieves embolization by entering the vessel and polymerizing with cations, thereby occluding the vessel lumen [3]. Unlike a gelatin sponge, NBCA is a permanent embolization material.

Several studies have reported that NBCA for PPH with DIC is effective [21–23]. However, the number of these reports is limited. Complications, including the effect of NBCA on future pregnancies, are also unclear. Additionally, this technique requires specific skills. Therefore, we consider that the use of NBCA should be restricted until further evidence is provided.

In a recent review, prior UAE was associated with PPH and the placenta previa spectrum during the next pregnancy [24]. UAE may reduce blood flow to the uterus and damage the endometrium. Additionally, repeated UAE may be associated with higher complication rates because the use of a large volume of embolic agents blocks blood flow in the uterus and damages it [24].

**Fig. 4:**
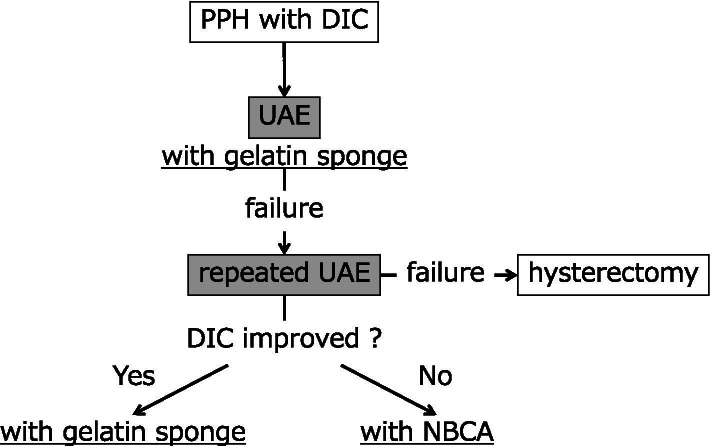
Strategy of repeated UAE for PPH with DIC. DIC: disseminated intravascular coagulation, NBCA: N-butyl cyanoacrylate, PPH: postpartum hemorrhage, UAE: uterine artery embolization

We propose the strategy of repeated UAE for PPH with DIC **(**Fig. 4**)**. UAE using a gelatin sponge is initially applied. If further bleeding occurs, repeated UAE using a gelatin sponge will probably achieve hemostasis if the DIC has improved, as in the current case. However, NBCA may be considered if the patient’s DIC has not improved. A recent review showed that UAE was beneficial for reducing blood loss and the operation time compared with peripartum hysterectomy, and it had a comparable hemostatic efficacy rate [25]. We recommend using repeated UAE before hysterectomy.

In conclusion, the success rate of UAE is reduced in patients with PPH with DIC, and requires careful consideration. Repeated UAE is an effective procedure and should help to avoid a hysterectomy.

## Data Availability

All data related to this report are available from the corresponding author on reasonable request.

## Abbreviations

DIC: disseminated intravascular coagulation
FDP: fibrin degradation product
FFP: fresh frozen plasma
ISTH: International Society for Thrombosis and Hemostasis
NBCA: N-butyl cyanoacrylate
PPH: postpartum hemorrhage
RBCs: red blood cells
UAE: uterine artery embolization
